# Inactivation of the Anterior Cingulate Reveals Enhanced Reliance on Cortical Networks for Remote Spatial Memory Retrieval after Sequential Memory Processing

**DOI:** 10.1371/journal.pone.0108711

**Published:** 2014-10-03

**Authors:** Brianne C. Wartman, Jennifer Gabel, Matthew R. Holahan

**Affiliations:** Department of Neuroscience, Carleton University, Ottawa, Ontario, Canada; University of Lethbridge, Canada

## Abstract

One system consolidation model suggests that as time passes, ensembles of cortical neurons form strong connections to represent remote memories. In this model, the anterior cingulate cortex (ACC) serves as a cortical region that represents remote memories. However, there is debate as to whether remote spatial memories go through this systems consolidation process and come to rely on the ACC. The present experiment examined whether increasing the processing demand on the hippocampus, by sequential training on two spatial tasks, would more fully engage the ACC during retrieval of a remote spatial memory. In this scenario, inactivation of the ACC at a remote time point was hypothesized to produce a severe memory deficit if rats had been trained on two, sequential spatial tasks. Rats were trained on a water maze (WM) task only or a WM task followed by a radial arm maze task. A WM probe test was given recently or remotely to all rats. Prior to the probe test, rats received an injection of saline or muscimol into the ACC. A subtle deficit in probe performance was found at the remote time point in the group trained on only one spatial task and treated with muscimol. In the group trained on two spatial tasks and treated with muscimol, a subtle deficit in probe performance was noted at the recent time point and a substantial deficit in probe performance was observed at the remote time point. c-Fos labeling in the hippocampus revealed more labeling in the CA1 region in all remotely tested groups than recently tested groups. Findings suggest that spatial remote memories come to rely more fully on the ACC when hippocampal processing requirements are increased. Results also suggest continued involvement of the hippocampus in spatial memory retrieval along with a progressive strengthening of cortical connections as time progresses.

## Introduction

Memory consolidation is a naturally occurring process whereby recently encoded memory representations become more resistant to decay over time. Consolidation processes apply to neural modifications that happen both at the cellular and systems levels [1]–[6]. Systems consolidation is a gradual process, occurring over days to years. One hypothesis suggests that memories may come to be represented by neural circuits that are linked to, but independent from, the neural ensembles that initially encoded and temporarily represented the memory. In this view, memory representations come to rely on distinct neural ensembles from those that initially encoded the memory for remote storage [4], [7]–[11].

One brain structure that critically contributes to memory function is the hippocampus [12]–[16]. Largely, though not exclusively, human research has shown that damage to the hippocampus results in temporally graded retrograde amnesia such that memories from the recent past are lost but memories from the remote past are spared [17]–[21]. This supports the hypothesis that memory representations initially encoded by the hippocampus become disengaged from those circuits over time for remote storage. Hippocampal damage in animal models has been reported by some to result in temporally graded retrograde amnesia [10], [22]–[25] but others have reported flat, or ungraded, retrograde amnesia following hippocampal damage [26]–[37], such that memories from both the recent and remote past are disrupted.

One cortical region that may be involved in the storage of remote memories is the anterior cingulate cortex (ACC). Reports have shown increased ACC activity on tests for remote memory (∼30 d after encoding) [10], [25], [38]–[41] and structural changes, indicative of memory storage, within the ACC have been observed at remote time points [42], [43]. In this framework, neural ensembles within the ACC would come to support memories for remote storage even in the absence of their involvement in initial memory encoding (see [2], [44] for discussion).

A number of studies have shown that inactivation of the ACC hinders performance on remote memory tests based on spatial tasks or tasks that involved spatial exploration [10], [25], [39], [40], [45], [46]. The present experiment examined whether a memory would more fully come to rely on neural ensembles in the ACC when distinct memories were sequentially encoded by overlapping neural ensembles in the hippocampus. Based on this, it was hypothesized that inactivation of the ACC would more fully disrupt remote memory retrieval in rats that had been trained on two sequential spatial tasks, the water and radial arm maze, as opposed to one. Both of these tasks rely on hippocampal neural ensembles for encoding so it might be the case that the ACC becomes fully engaged in remote memory storage when hippocampal demand is taxed during early phases of the consolidation process.

## Materials and Methods

### Subjects

Sixty-four (64) male Long Evans rats (190–250 g) from Charles River, Quebec were used. Rats were housed individually in clear plastic cages (26×20×45 cm) and given ad libitum water under a 12-h light/dark cycle (lights on at 8:00 a.m.; rats tested during the light phase). Rats received no nesting material and no direct enrichment of any kind in their home cage. Rats acclimated to the vivarium environment for a minimum of five days prior to the beginning of experimental procedures. Principles of laboratory animal care were followed and all procedures were conducted in accordance with the Canadian Council on Animal Care and protocols approved by the Carleton University Animal Care Committee.

### Surgical Procedures

Rats were administered Isofluorane gas anesthesia (3% in pure 0_2_ to induce and 2% in pure O_2_ to maintain). Rats were defined to have reached an appropriate level of anesthesia when there was a loss of toe-pinch and pedal reflexes. This reflex was checked throughout surgical procedures. The head was shaved and rats were mounted in a stereotaxic apparatus. The scalp was cleaned and disinfected using betadine. Because the surgical procedures typically lasted approximately 45 minutes, a lubricating ophthalmic ointment (tear gel) was applied to the eyes of each rat. A midline incision in the scalp was made from behind the eyes to the ears using a 10 mm scalpel. Skin was scraped from the skull and hemostats were used to hold the incision open. Rats were bilaterally implanted with stainless steel 10 mm guide cannulas (25gauge) into the anterior cingulate cortex (ACC). With the tooth bar set at −3.9 mm [47] cannula tips were placed at coordinates (Anteroposterior: +1.2; Mediolateral: +/−0.5; Dorsoventral: −2.0). Removable stylets (32 gauge) were inserted into the guide cannula to keep them free from blockage. The cannulae were anchored to the skull with screws and dental cement. Polysporin was applied to the surgical area and the incision was closed using sutures. Topical lidocaine was applied to the incision site and a subcutaneous injection of Metacam (0.1 mL) was given. Rats were returned to their home cage, which was placed on a heating pad set to low. Soft food and water were placed inside the cage. Rats were under close surveillance until they were fully ambulatory. Basic biological functions including food and water intake, urination, defecation and body weight as well as clinical signs of distress (e.g., piloerection, reduced locomotion, hypothermia) were monitored daily. The surgical site and wound were monitored routinely for signs of infection. Rats recovered from surgery for a minimum of 7 days before further experimental procedures began.

### Apparatus

#### Water Maze (WM)

The WM was located in a room within the animal housing area. The opaque, white, polypropylene pool measured 155 cm in diameter and 60 cm in height. The pool was filled to a depth of 37.5 cm with water that remained at approximately 21°C. The ‘escape’ platform was made from clear Plexiglas and submerged approximately 2 cm below the surface of the water. Visual cues such as posters and geometric shapes were located on the walls around the room. The experimenter remained in the same position throughout all trials.

**Radial Arm Maze (RAM).** RAM testing was done in a room within the animal housing area, located across the hall from the WM testing room. The maze was positioned 98.5 cm off the floor. Each arm measured 59 cm long and 11 cm wide. The distance between the ends of arms, where food (chocolate pellet, BioServe, New Jersey) reward was located, was 32.5 cm. Plastic inserts were placed on the sides of the maze arms to prevent animals from jumping across arms. Visual cues such as posters and geometric shapes were located on the walls around the room. The experimenter remained in the same position throughout all trials.

### Behavioural Procedure

**Figure 1:**
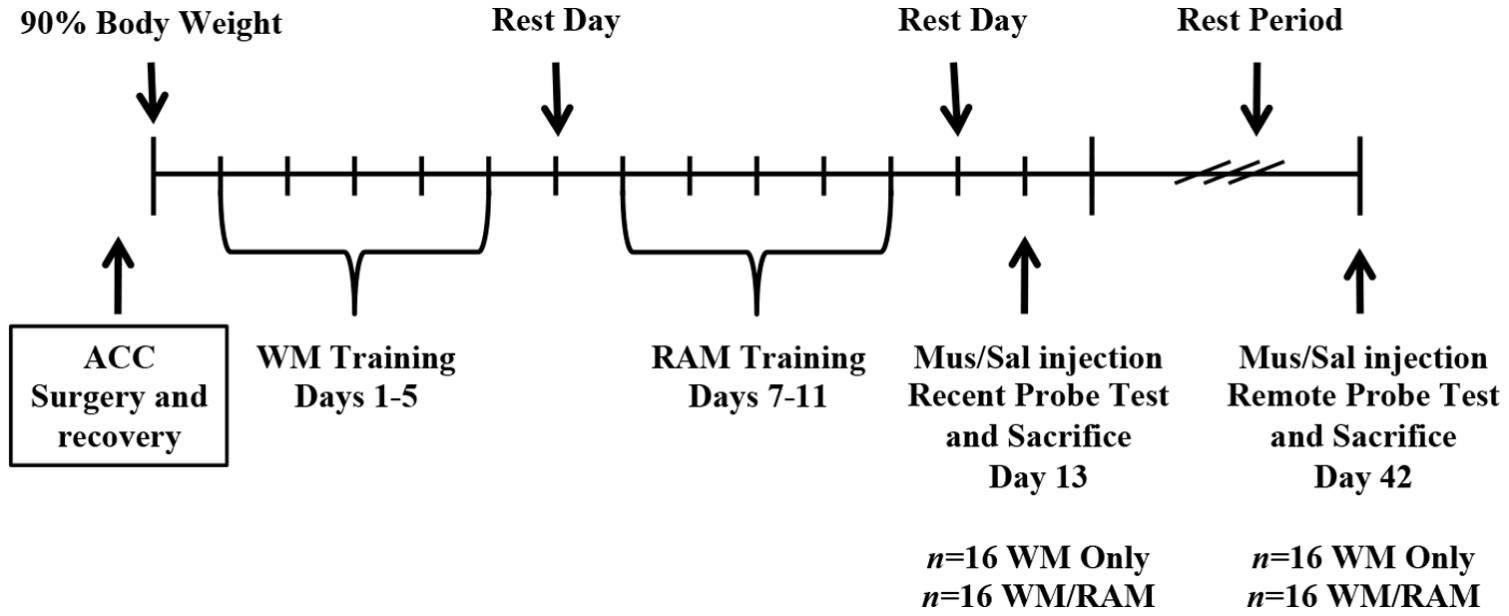
Timeline of experimental procedures.

An overview of the behavioural procedure is shown in Fig. 1. Seven days following surgery, rats were food restricted to 90% of their free feeding body weight over a minimum of 10 days. Rats were assigned to one of two behavioural groups: (1) Training on one spatial task, the WM; or (2) Training on two spatial tasks, the WM followed by the RAM. Sequential training on two tasks was separated by a 24-h rest period. Following training, rats received either a recent WM probe test (8 days after the end of WM training, *n* = 32) or a remote WM probe test (37 days after the end of WM training, *n* = 32). Additionally, 15 minutes prior to the probe test, rats were assigned to receive an intracranial injection of muscimol, a GABA_A_ receptor agonist, or saline. Eight experimental groups resulted (*n* = 8 in each group): (1) WM:Recent:Saline (2) WM:Recent:Muscimol (3) WM/RAM:Recent:Saline (4) WM/RAM:Recent:Muscimol (5) WM:Remote:Saline (6)WM:Remote:Muscimol (7) WM/RAM:Remote:Saline (8) WM/RAM:Remote:Muscimol.

#### Water maze

Rats received 5 training trials per day for 5 days, with a different starting location for every trial within a day and randomized starting locations across days. The hidden platform was located in a fixed location within and across days. Rats were placed in the pool, facing the perimeter, and given a maximum of 60 s to locate the hidden platform. Rats that did not find the hidden platform within 60 s were guided to the platform by the experimenter. All rats remained on the platform for 15 s. Rats then received a 15 s rest period in a holding cage before the next trial. All movement within the pool was tracked using HVS Image 2100 Tracking System (HVS Image, Buckingham, UK). Following the final trial of each day, rats were dried with a towel and placed in a holding cage on a heating pad in the housing room for 10–15 min after which they were returned to the home cage.

#### Radial arm maze

Rats received one day of pretraining and 4 days of testing on the RAM. On the first trial of pretraining, chocolate pellets were located in the starting area, at the entrance to arms, within the arms as well as in the food holes located at the end of each arm. On trial 2 of pretraining, pellets were located within the arms and in food holes only. Trials 3–5 of pretraining had pellets located only in food holes.

Days 2–5 were testing days, where pellets were located in food holes at the ends of 3 of the 5 arms. Baited arms were always the same for an individual rat but differed between rats. Each rat was given 5 trials per day. Trials were a maximum of 5 min each or ended when all food reward had been collected. Rats were placed in a holding cage for 30 s between trials while arms were re-baited. Performance on the maze was manually scored. Sessions were timed and correct and incorrect arm entries were recorded. An arm entry was defined as all four feet inside an arm.

### Injections

Rats were handled daily throughout experimental procedures and rest periods to minimize stress during testing and injection procedures. Injections were performed on awake rats, held by the experimenter for the duration. Stylets were removed and two stainless-steel injection cannula (32 gauge), connected to two individual 10 µL Hamilton syringes via polyethylene tubing, were carefully lowered through the guide cannula. The syringes were connected to an injection pump (Braintree Scientific, Inc.). The pump was programmed to deliver a volume of 0.5 µL of muscimol (50 ng/0.5 µL) or saline solution at a rate of 0.25 µL/min, with a total infusion time of two minutes. The injection cannulae were left in place for an additional one minute. The injectors were removed and rats were placed in their home cage for 15 min before probe testing.

### Probe Test

The escape platform was removed from the water maze pool for the probe test. Rats were placed in the pool and free to swim for 60 s. Rats were then removed from the pool, dried with a towel, placed on a heating pad and brought back to the home cage room. Sixty minutes after the end of the WM probe test, rats were placed into a Decapicone (Braintree) and decapitated. Brains were rapidly removed and hemisected (hemispheres were counterbalanced). One hemisphere was placed in 4% paraformaldehyde/0.01 M phosphate buffer solution (PB; pH 7.4) overnight at 4°C. The following day brains were cryoprotected in a 30% sucrose/0.1 M PB solution and stored at 4°C for a minimum of 72 h or until sectioned.

### Cresyl Violet

Brains were sectioned at 30 µm on a Leica CM1900 cryostat (Weztler, Germany). Sections were stored in a 0.1% sodium azide/0.1 M PB solution at 4°C. Sections containing the ACC were float mounted on microscope slides and placed in 100%, 95%, and 70% ethanol solutions for two minutes each. Excess ethanol was removed with a rinse in distilled water. Sections were then placed in a 1% Cresyl Violet solution for three minutes. Excess stain was removed with a rinse in distilled water. Sections were place in a 0.8% acetic acid solution until fiber tracks became unstained (approximately 3–5 min). Sections were placed in 70%, 95% and 100% ethanol solutions for two minutes, followed by a 15 min incubation in Clearene. Slides were coverslipped using Permount mounting medium. Once dry, sections were examined under a light microscope to verify cannula placements.

### c-Fos immunohistochemistry

Sections were washed for 3×5 min in a 0.2% Triton-X/0.01 M phosphate-buffered saline (T-PBS) solution then blocked for 15 min in a 0.3% H_2_O_2_ T-PBS solution. Sections were washed for 3×5 min in T-PBS solution then blocked in a 3% Animal Free Blocker (AFB) (Vector)/T-PBS solution for 30 min followed by incubation in the primary antibody (rabbit anti-c-Fos from Abcam, 1∶5000) overnight at room temperature. The following day, sections were washed for 3×10 min in T-PBS solution then incubated for two hours in the secondary antibody (biotinylated goat anti-rabbit, 1∶500). Sections were then washed 3×10 min in T-PBS followed by one-hour incubation in an avidin-biotinylated complex (ABC Elite kit; Vector Laboratories). Sections were given a wash in PBS then reacted with a 0.25% 3, 3′-diaminobenzedine tetrahydrochloride (DAB) solution in PBS solution containing 0.0025% H_2_O_2_ for 6 min. All sections were given a final rinse in 0.01 M PBS for 15 min and mounted on glass slides. Sections were dehydrated and coverslipped with glass coverslips and Permount (Sigma).

### c-Fos quantification

The Optical Fractionator method was used to provide an estimate of the number of c-Fos positive cells in the CA1 region of the dorsal hippocampus. Stained sections were visualized using an Olympus BX51 brightfield microscope with a motorized stage (Olympus Canada, Markham, ON) and images captured with an Olympus U-CMAD3 camera. Stereo Investigator (MBF Bioscience, Williston, VT) software was used for quantification. The CA1 volume of interest focused on the anterior aspect of the dorsal hippocampus and was restricted in the anterior-posterior plane from bregma −3.3 to −3.6 mm. The region of interest for each section was traced digitally at 4× magnification with reference to [47]. Two to three random coronal sections were sampled from this region from each rat. Similar to unbiased stereological quantification methods, c-Fos counting was performed using sampling parameters sufficient to produce a Gunderson's coefficient of error (GCE, *m* = 1) less than 0.1, which has been established to be a suitable coefficient of error estimate [48]–[50]. A minimum of 6 animals per group were included in the analysis. Counting parameters were set to a counting frame of 30×30 µm^2^ and a dissector height of 10 µm between the top and bottom guard zones. c-Fos positive cells were quantified using a 60× magnification lens (oil immersion, NA 1.35) when the uppermost tip of c-Fos positive nuclei were in focus within the counting frame and the dissector height. Stereo Investigator software used planar and depth information for each counted nuclei to calculate the volume for the digitally traced region of interest. Quantification is represented as an estimated total per mean measured thickness per 10,000 µm^3^ to allow for comparisons across brain sections.

## Results

### Behavioural training

A timeline of the behavioural procedure is shown in Figure 1. Rats were randomly assigned to one of two behavioural groups: (1) Training on one spatial task, the WM or (2) Training on two spatial tasks, the WM followed by the RAM. Sequential training on two tasks was separated by a 24-h rest period. Following training, rats received either a recent WM probe test (8 days after the end of WM training, *n* = 32) or a remote WM probe test (37 days after the end of WM training, *n* = 32). Fifteen minutes prior to the probe test, rats were assigned to receive an intracranial injection of muscimol, a GABA_A_ receptor agonist, or saline into the ACC. Eight experimental groups resulted (*n* = 8/group): (1) WM:Recent:Saline (2) WM:Recent:Muscimol (3) WM/RAM:Recent:Saline (4) WM/RAM:Recent:Muscimol (5) WM:Remote:Saline (6)WM:Remote:Muscimol (7) WM/RAM:Remote:Saline (8) WM/RAM:Remote:Muscimol.

**Figure 2:**
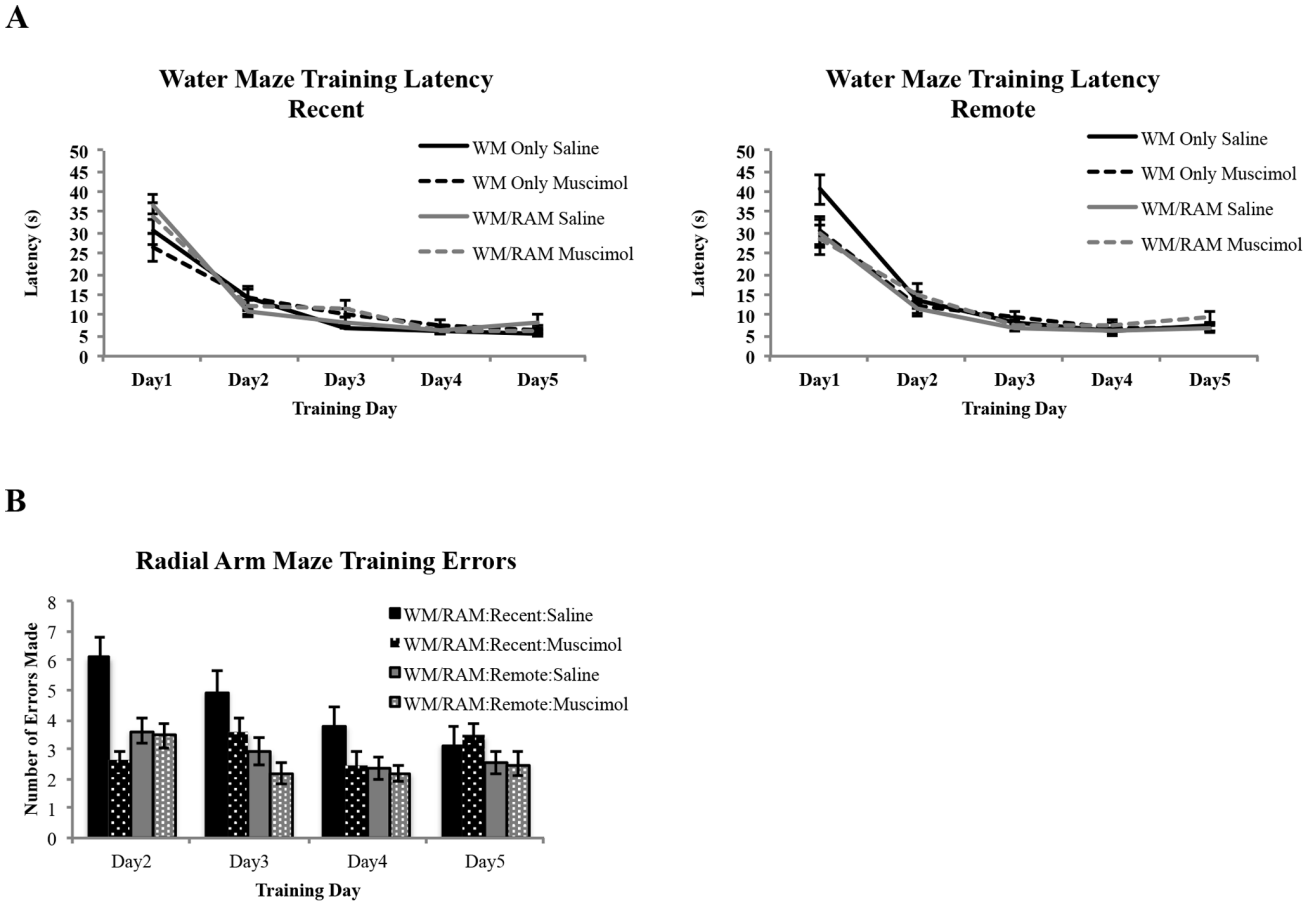
Behavioural training data from rats to-be assigned to different behavioural groups. A) Average latencies to reach the hidden platform during the five days of water maze training. Recent and remote time points are plotted and examined separately to reduce visual noise. At the recent time point, a main effect of day was found. Latency to reach the hidden platform decreased across training day indicating an improvement in performance. No main effect of behaviour or treatment was found. Similarly, at the remote time point a main effect of day was found. Latency to reach the hidden platform decreased across training day indicating an improvement in performance. No main effect of behaviour or treatment was found. B) Average number of errors (unbaited arm entries and re-entries) made in the radial arm maze task during the four days of training. A main effect of day was found. Errors made decreased across training day indicating an improvement in performance. No main effect of time or treatment was found.

Figure 2 shows acquisition data for both the WM and RAM tasks. All rats received WM training and group designation, as shown in Figure 2, is based on manipulation prior to the probe test. Latency to reach the hidden platform was recorded (Fig. 2A). To reduce visual noise, recent and remote time points were plotted and examined separately. At the recent time point, a repeated measures ANOVA with day (1 to 5) as the within-subject factor and behaviour (WM Only or WM/RAM) and treatment (Saline or Muscimol) as the between-subjects factors revealed a main effect of day (F(4,112) = 73.14, p<0.001) but no main effect of behaviour (F(1,28) = 0.94, p>0.05) or treatment (F(1,28) = 0.001, p>0.05). At the remote time point, a repeated measures ANOVA with day (1 to 5) as the within-subject factor and behaviour (WM Only or WM/RAM) and treatment (Saline or Muscimol) as the between-subjects factors revealed a main effect of day (F(4,112) = 140.28, p<0.001) but no main effect of behaviour (F(1,28) = 1.56, p>0.05) or treatment (F(1,28) = 0.06, p>0.05).

Four groups received training on the WM followed by training on the RAM: one assigned to receive a recent WM probe test (Saline or Muscimol) and one assigned to receive a remote WM probe (Saline or Muscimol). The number of errors (unbaited arm entries and re-entries) made during training (Fig. 2B) was analyzed with a repeated measures ANOVA with day (2 to 4) as the within-subject factor and time (recent or remote) and treatment (saline or muscimol) as the between-subject factors. Analyses revealed a main effect of day (F(3,84) = 7.85, p<0.001) but no main effect or time (F(1,28) = 2.53, p>0.05) or treatment (1,28) = 1.775, p>0.05).

### Water maze probe test

To provide an accurate depiction of the search patterns exhibited by the different groups during the probe test, data were examined for the entire 60 second probe test and also parsed into two time bins: from 0–30 seconds and 31–60 seconds. This analysis allowed us to examine whether the initial search patterns (0–30 s) focused on the correct area of the pool while later search patterns (31–60 s) expanded to other areas of the pool or remained in the correct (target) area of the pool. Raw data for all probe test data from all groups used in the present experiment are included in Data S1.

Water maze probe data were examined in terms of time spent swimming in the target quadrant and target area. The quadrant represents a large area of the WM pool (155 cm diameter; one quadrant  = 25%; chance performance  = 25%) compared to the size of the platform (11 cm diameter). The target area represents the platform region more accurately (chance performance  = 2%). We operationally defined a severe memory deficit as a lack of preference for the target quadrant and a slight memory deficit as a lack of preference for the target area.

#### Surface Plot

**Figure 3:**
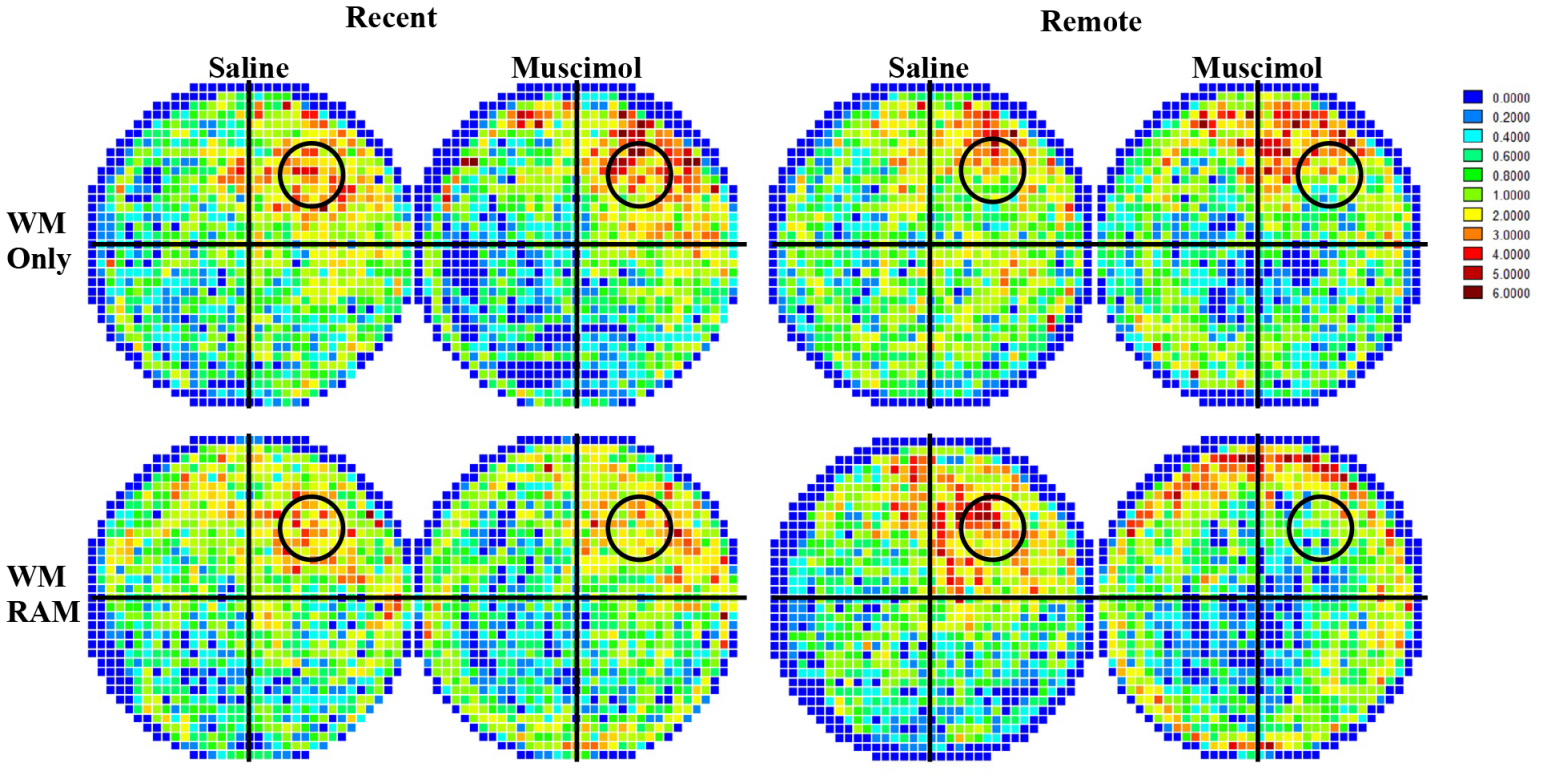
Average dwell times in discrete areas of the pool for all eight experimental groups during the 60 second water maze probe test. The scale bar colors represent average time spent in an area of the pool derived from group data. The circle represents the location of the hidden platform during training.

Average dwell times in discrete regions of the water maze for the eight groups over the 60 second water maze probe test are illustrated in Figure 3 as surface or occupancy plots generated from Wintrack software [51]. The surface plots show a clear preference in search strategy for the area associated with the platform location (circle) in groups WM Only:Recent:Saline, WM Only:Recent:Muscimol, WM Only:Remote:Saline WM/RAM:Recent Saline, and WM/RAM:Remote Saline. Groups WM Only:Remote:Muscimol and WM/RAM:Recent:Muscimol showed less accurate search patterns for the area associated with the platform location. A clear reduction in dwell time in the platform location can be seen in group WM/RAM:Remote:Muscimol.

#### Target quadrant

Rats received either a recent or remote WM probe test. The amount of time spent swimming in the target quadrant (quadrant where the platform was located during training) was examined. Separate 2×2×2 (behaviour (WM Only or WM/RAM) × time (recent or remote) × treatment (saline or muscimol) fixed factor ANOVAs were run.

**Figure 4:**
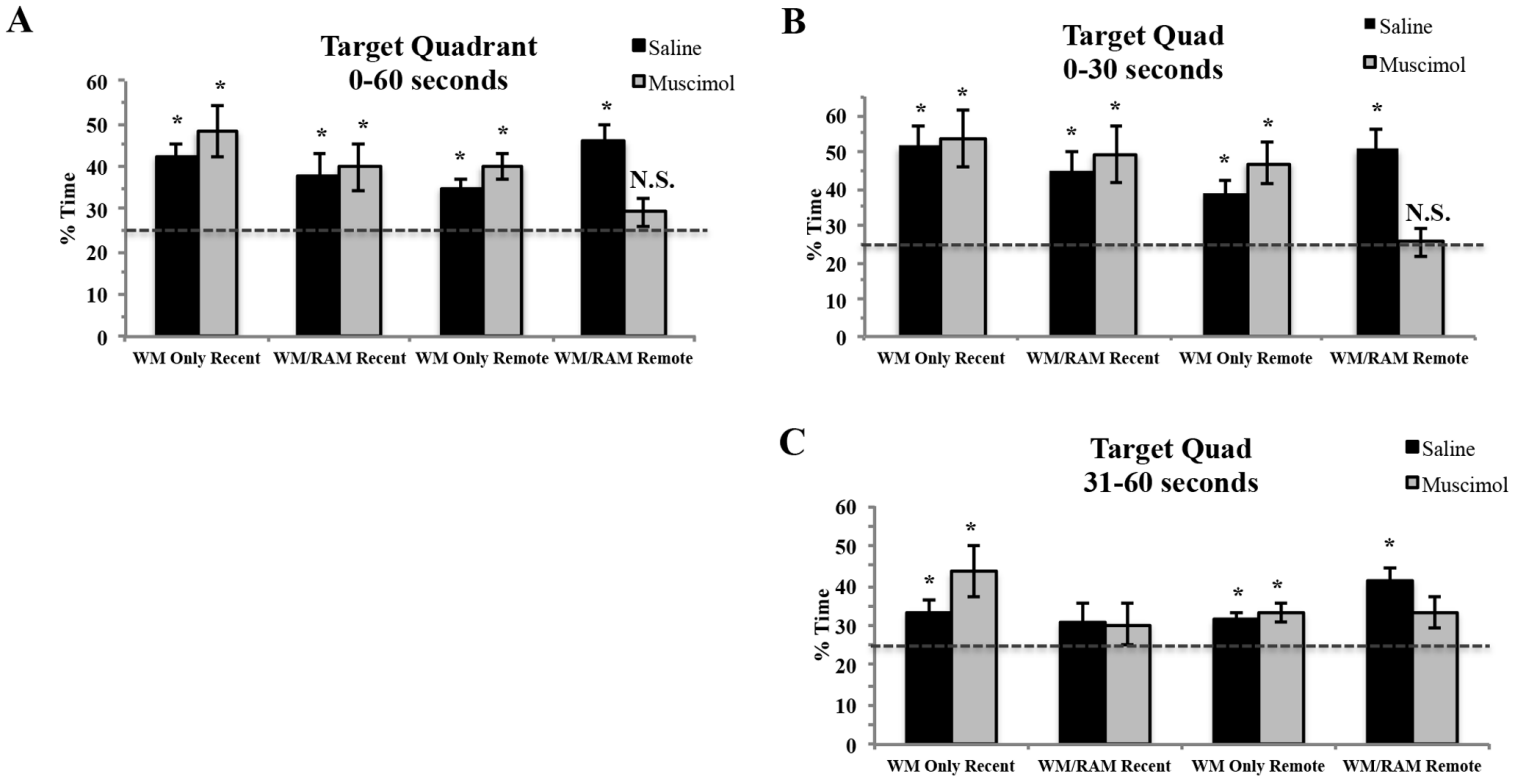
Percent of time spent swimming in the target quadrant during the 60 second probe (A), from 0–30 seconds (B) and 31–60 seconds (C). The dashed line represents chance performance (25%). A) During the 60 second probe, all experimental groups spent above chance amounts of time swimming in the target quadrant, except rats trained on two spatial tasks, tested remotely and given a muscimol injection (WM/RAM:Remote:Muscimol). B) From 0–30 seconds, all experimental groups spent above chance amounts of time swimming in the target quadrant, except rats trained on two spatial tasks, tested remotely and given a muscimol injection (WM/RAM:Remote:Muscimol). C) From 31–60 seconds fewer groups displayed a preference for the target quadrant. * Significantly different from chance performance (p<0.05).

Statistical analyses on time spent in the target quadrant during the 60 second probe (Fig. 4A) revealed no main effect of behaviour (F(1,56) = 1.14, p>0.05), time (F(1,56) = 2.39, p>0.05) or treatment (F(1,56) = 0.11, p>0.05). A significant behaviour × treatment interaction was found (F(1,56) = 4.48, p<0.05). When time spent searching in the target quadrant was compared to chance (25%), all experimental groups spent above chance amounts of time searching in the target quadrant except group WM/RAM:Remote:Muscimol (p>0.05).

Statistical analyses on time spent in the target quadrant from 0–30 seconds (Fig. 4B) revealed a main effect of time (F(1,56) = 5.40, p = 0.02) with recent groups spending greater amounts of time in the target quadrant compared to remote groups (Recent mean: 49.90% Remote mean: 40.53%). A significant interaction between behavior and treatment (F(1,56) = 3.73, p = 0.05), and a significant three-way interaction between behavior, time and treatment (F(1,56) = 4.82, p = 0.03) were found. When time spent searching in the target quadrant was compared to chance (25%), all experimental groups spent above chance amounts of time searching in the target quadrant during the first 30 seconds of the probe test except group WM/RAM:Remote:Muscimol (p>0.05).

Statistical analyses on time spent in the target quadrant from 31–60 seconds (Fig. 4C) revealed fewer groups spent above chance amounts of time searching in the target quadrant (WM Only:Recent:Saline, WM Only:Recent:Muscimol, WM Only:Remote:Saline, WM Only:Remote:Muscimol and WM/RAM:Remote:Saline spent above chance amounts of time searching in the target quadrant). A significant interaction between behavior and time (F(1,56) = 4.35, p = 0.04) was found, but no other significant effects or interactions were noted (behaviour (F(1,56) = 0.22, p>0.05), time (F(1,56) = 0.01, p>0.05), treatment (F(1,56) =  0.1, p>0.05)).

#### Target counter region

The amount of time spent swimming in the target area (a circular region around the previous platform position, 2× the radius; referred to as “counter” in the parlance of the HVS Image software) was examined. Separate 2×2×2 (behaviour (WM Only or WM/RAM) × time (recent or remote) × treatment (saline or muscimol)) fixed factor ANOVAs were run.

**Figure 5:**
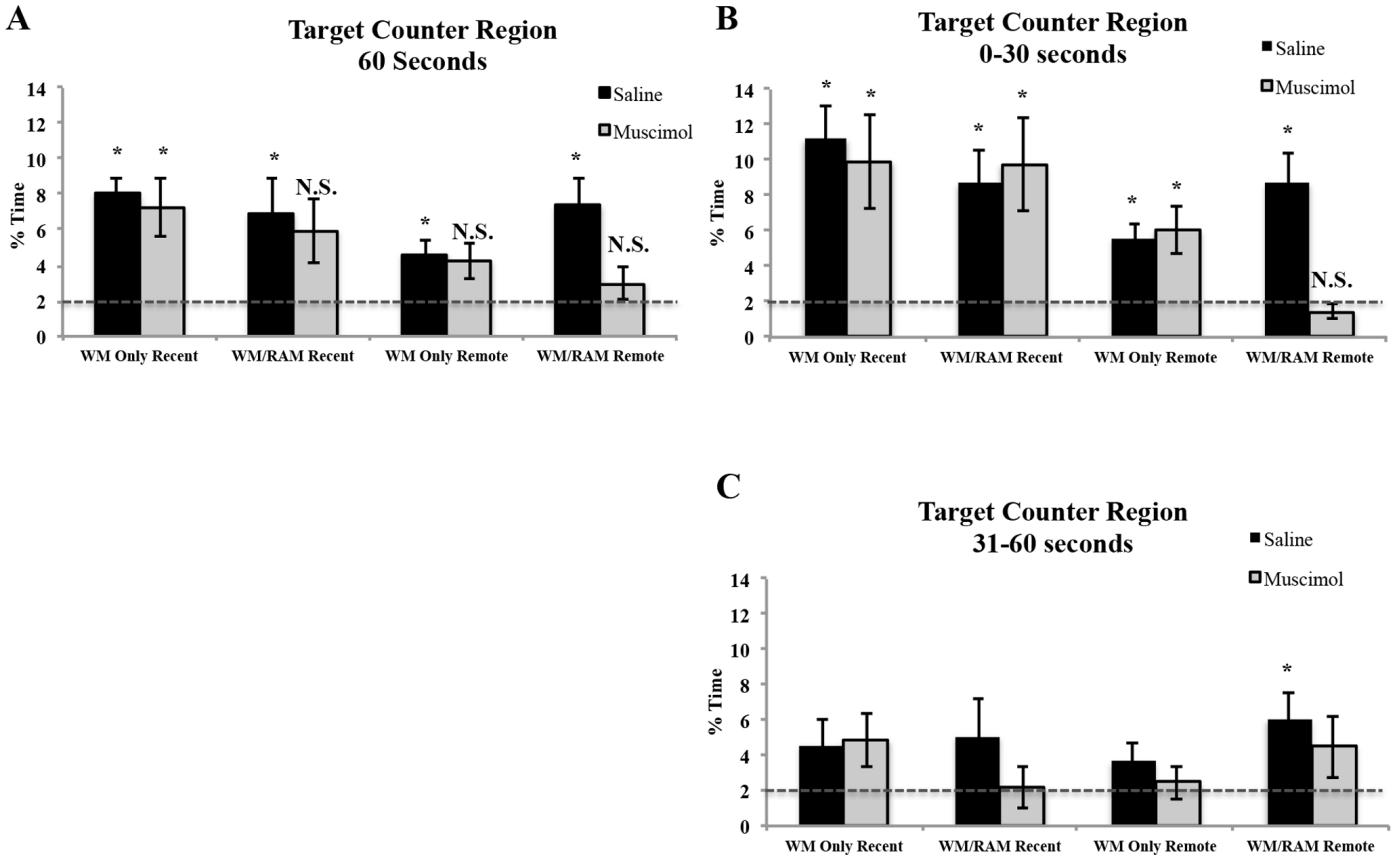
Percent of time spent swimming in the target area during the 60 second probe (A), from 0–30 seconds (B) and 31–60 seconds (C). The dashed line represents chance performance (2%). A) During the 60 second probe, all experimental groups spent above chance amounts of time swimming in the target area, except the group trained on two spatial tasks, tested remotely and given a muscimol injection (WM/RAM:Remote:Muscimol), as well as groups WM Only:Recent:Muscimol and WM/RAM:Recent:Muscimol. B) From 0–30 seconds, all experimental groups spent above chance amounts of time swimming in the target area, except rats trained on two spatial tasks, tested remotely and given a muscimol injection (WM/RAM:Remote:Muscimol). C) From 31–60 seconds, only group WM/RAM:Remote:Saline spent above chance amounts of time in the target area. * Significantly different from chance performance (p<0.05).

Statistical analyses on time spent in the target area during the 60 second probe (Fig. 5A) revealed a main effect of time (F(1,56) = 5.27, p<0.05), with recently probed rats spending greater amounts of time in the target area (7.05%) compared to remotely probed rats (4.81%). When time spent searching in the target area was compared to chance (2%), experimental groups WM Only:Recent:Saline, WM Only:Recent:Muscimol, WM/RAM:Recent:Saline, WM Only:Remote:Saline and WM/RAM:Remote:Saline spent above chance amounts of time searching in the target area (p<0.05). Groups WM/RAM:Recent:Muscimol, WM: Only:Remote:Muscimol and WM/RAM:Remote:Muscimol did not spent above chance amounts of time searching in the target area (p>0.05).

Statistical analyses on time spent in the target area from 0–30 seconds (Fig. 5B) revealed a main effect of time (F(1,56) = 11.82, p<0.001) with recently probed rats spending a greater amount of time in the target area compared to remotely probed rats (Recent mean: 9.91% Remote mean: 5.44%). A significant three-way interaction between behaviour, time and treatment (F(1,56) = 3.77, p = 0.05) was found. Fisher's LSD post hoc tests revealed group WM/RAM:Remote:Muscimol spent less time swimming in the target area compared to all other groups (except WM Only:Remote:Saline (p = 0.11) and WM Only:Remote: Muscimol (p = 0.08)). When the time spent searching in the target area was compared to chance (2%) all experimental groups spent above chance amounts of time in the target area during the first 30 seconds of the probe test except group WM/RAM:Remote:Muscimol (p>0.05).

Statistical analyses on time spent in the target counter region from 31–60 seconds (Fig. 5C) revealed only group WM/RAM:Remote:Saline spent above chance amounts of time searching in the target area. No other main effects or significant differences between experimental groups were found (behaviour (F(1,56) = 1.61, p>0.05), time (1,56) = 0.92, p>0.05), treatment (F(1,56) = 0.49, p>0.05)).

#### Target Area Crossings

The number of crosses through the target area (2× the diameter of the platform) was examined. Separate 2×2×2 (behaviour (WM Only or WM/RAM) × time (recent or remote) × treatment (saline or muscimol)) fixed factor ANOVAs were run. Comparisons were made across groups as no chance level of performance exists.

**Figure 6:**
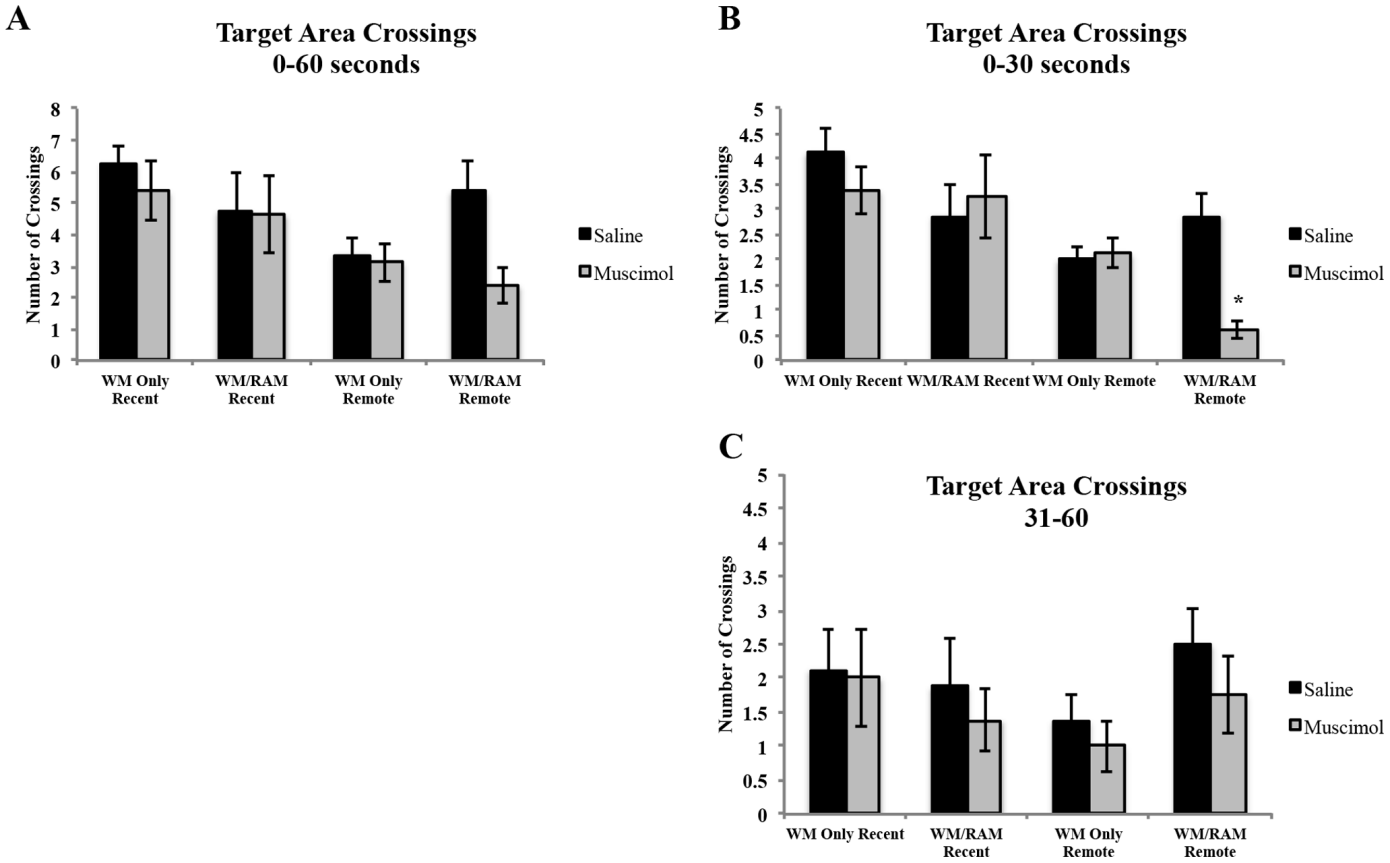
Number of crossings through the target area during the 60 second probe A), from 0–30 seconds (B) and 31–60 seconds (C) during the water maze probe test. A) During the 60 second probe, no significant differences between experimental groups were found. B) From 0–30 seconds, rats trained on two spatial tasks, tested remotely and given a muscimol injection (WM/RAM:Remote:Muscimol) had significantly fewer crossings through the target area compared to all other experimental groups. C) From 31–60 seconds, no significant differences between experimental groups were found. * Significantly different from all other experimental groups (p = 0.05).

Statistical analyses on the number of crossings during the 60 second probe (Fig. 6A) revealed a main effect of time (F(1,56) = 7.57, p<0.01) with recently tested rats having a greater number of crosses through the target area (5.25) compared to remotely tested rats (3.56).

Statistical analyses on the number of crossings from 0–30 seconds (Fig. 6B) revealed a main effect of time (F(1,56) = 18.88, p<0.001) with recently probed rats displaying a greater number of crossings through the target area (Recent mean: 3.41 Remote mean: 1.91). A significant three-way interaction between behaviour, time and treatment (F(1,56) = 6.43, p<0.02) was found. Fisher's LSD post hoc tests revealed group WM/RAM:Remote:Muscimol displayed fewer crossings through the target area during the first 30 seconds of the probe test compared to all other groups (p = 0.05).

Statistical analyses on the number of crossings through the target area from 31–60 seconds (Fig. 6C) revealed no main effects or significant differences between experimental groups (behaviour (F(1,56) = 0.41, p>0.05, time (F(1,56) = 0.23, p>0.05), treatment (F(1,56) = 1.25, p>0.05).

### c-Fos labeling in the CA1 of the hippocampus

**Figure 7:**
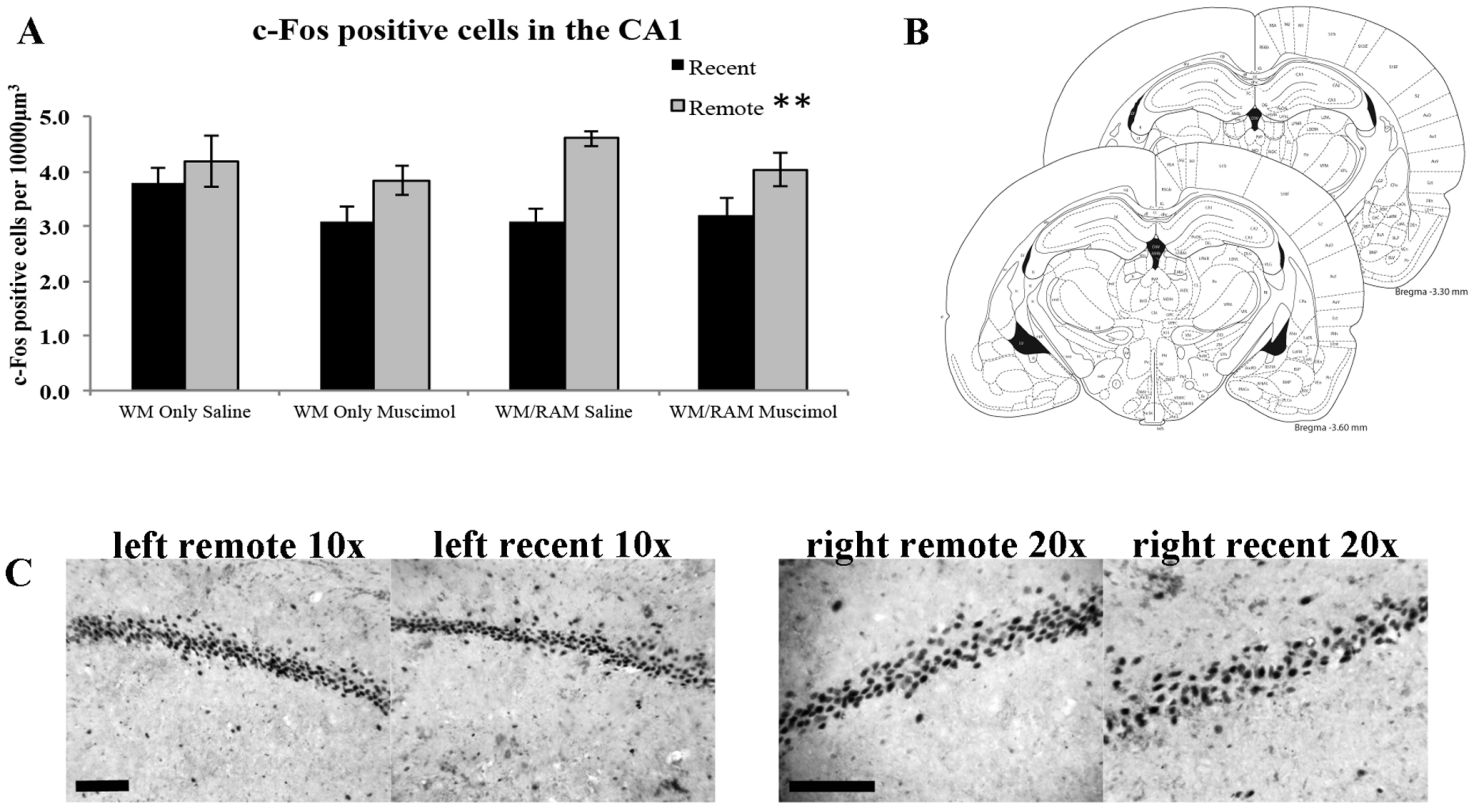
c-Fos positive cells in the CA1 of the hippocampus. A) Quantification of the number of c-Fos positive cells per 10000 µm^3^ in the CA1 of the hippocampus. Remotely probed rats displayed greater numbers of c-Fos positive cells than recently probed rats. B) Depiction of the CA1 area where c-Fos immunoreactive cells were counted (Paxinos & Watson, 1998). C) Representative images of c-Fos staining in the CA1 region in the left hemisphere at 10× and the right hemisphere at 20× magnification. Scale bar at both magnifications  = 100 µm. **Indicates a main effect of time (remote> recent).

Fig. 7A shows quantification of the number of c-Fos positive cells per 10,000 µm^3^ in the CA1 of the hippocampus. Fig. 7B depicts the CA1 region of the hippocampus where c-Fos positive cells were counted (spanning bregma −3.3 to −3.6). Fig. 7C displays representative images of c-Fos positive neurons at 10× and 20× magnifications from the left and right hemispheres from remote and recent groups, respectively. A 2×2×2 (behaviour (WM Only or WM/RAM) × time (recent or remote) × treatment (saline or muscimol) fixed factor ANOVA revealed a main effect of time (F(1,49) = 16.509, p<0.001) with remotely probed rats showing greater numbers of c-Fos positive cells compared to recently probed rats (Recent mean: 3.279 Remote mean: 4.157).

### Placements

**Figure 8:**
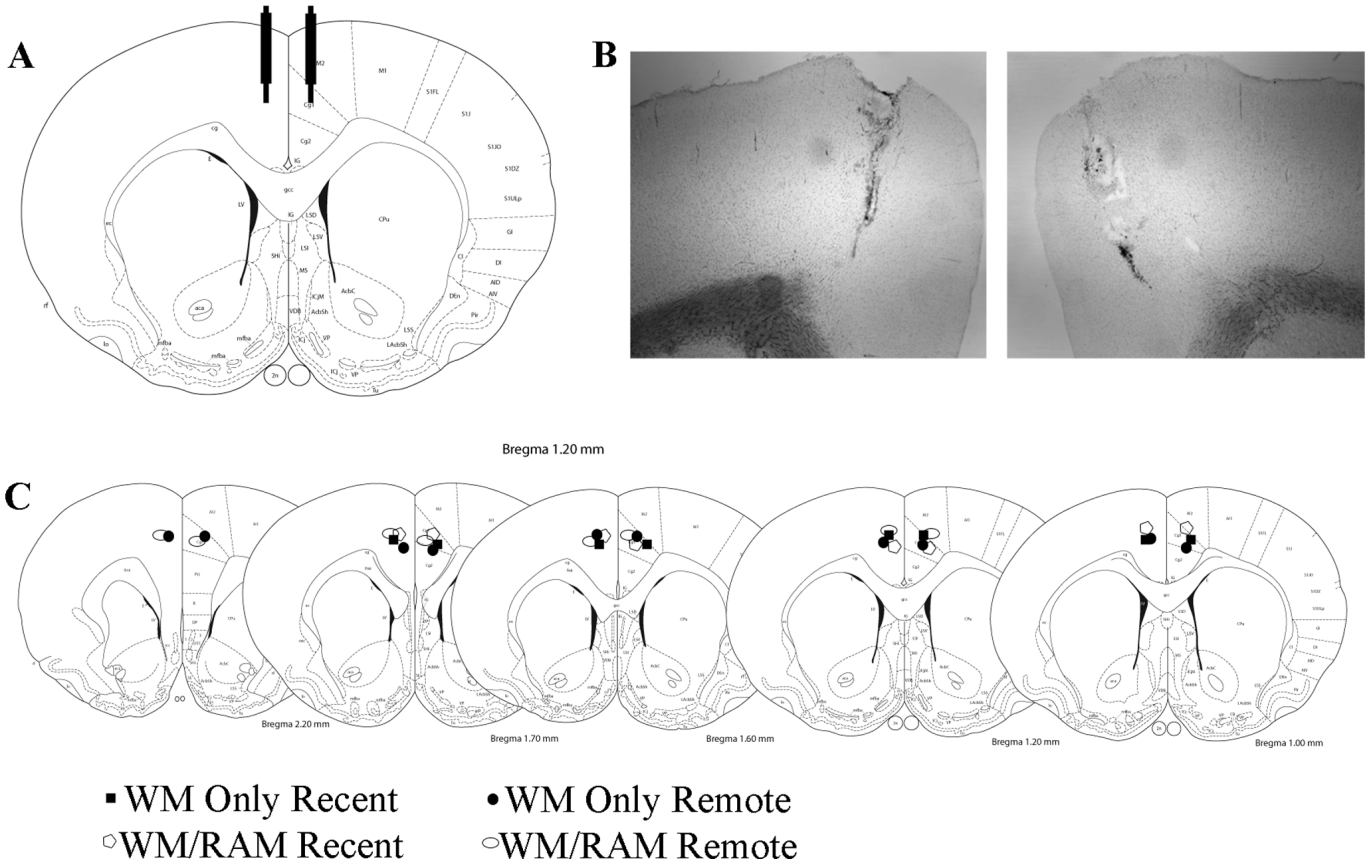
A) Schematic of intended cannula and injector placements based on stereotactic coordinates. B) Representative images of right and left hemisphere cannulation and injection sites (4× magnification). C) Approximate distribution of injection sites in the region of the left and right ACC from all groups (saline and muscimol injection sites are represented similarly).

Figures 8B and C show representative placements of injector tips aimed at the ACC. Postmortem analyses of cannula and injector placements confirmed consistent placement of injections into the ACC of all rats used. The range in injector tip locations was: Anteroposterior: +2.2 to +1.0; Mediolateral: +/− 0.2 to 0.7; Dorsoventral: −2.0 to −3.0 (Fig 8C). This range is well within the boundary of the ACC and, given prior estimates of the volume of inactivation produced by a similar volume and dose of muscimol [52], [53], the entire ACC was likely inactivated by the muscimol injections.

## Discussion

The purpose of this study was to investigate whether sequential training on two spatial tasks would more fully engage the ACC during retrieval of a remote spatial memory. Results revealed a substantial impairment in remote spatial memory recall in the group trained on two, sequential spatial tasks and injected with muscimol into the ACC prior to the remote probe test (WM/RAM:Remote:Muscimol). This group spent chance amounts of time in the target quadrant and target area and made fewer crossings through the target area compared to all other experimental groups. The control condition for this group (WM/RAM:Remote:Saline) showed a strong memory for the platform location during the probe test on all indices analyzed, emphasizing the profound memory deficit seen in group WM/RAM:Remote:Muscimol. The severely impaired performance in group WM/RAM:Remote:Muscimol supports the hypothesis that a remote spatial memory comes to rely more fully on neural ensembles in the ACC when distinct memories are sequentially encoded by overlapping neural ensembles in the hippocampus.

A slight deficit in memory recall was seen in groups WM Only:Remote:Muscimol and WM/RAM:Recent:Muscimol. The overall performance on the probe test revealed that these groups did not spend above chance amounts of time searching in the target area, indicative of a memory deficit. More stringent behavioural analyses revealed that these groups displayed above chance amounts of time in the target area during the first 30 seconds of the probe test, but dropped to chance amounts of time in the target area during the last 30 seconds of the probe test. The moderate impairment in group WM Only:Remote:Muscimol is in line with past research which has shown that the ACC becomes engaged in remote memory recall [10], [25], [39], [45], [46]. The slight impairment in group WM/RAM:Recent:Muscimol is consistent with our recent findings which suggested that taxing the demand placed upon the hippocampus, by training rats on two sequential spatial tasks, engaged the recruitment of the ACC at earlier time points [54].

The group trained on a single hippocampal-dependent task (WM Only) did not show evidence of reliance upon the ACC at the recent time point as there was no deficit in performance following muscimol treatment. Over time, the memory representation in the WM Only remote group came to partially rely on the ACC, as a slight deficit in probe performance was seen in this group following muscimol treatment. These results suggest the remote memory for the WM task in the WM Only group was not initially dependent upon the ACC but as time progressed, the memory may have become represented in neural ensembles in both the hippocampus and ACC. One factor that may be important in enhancing the dependence of a remote memory representation on ACC networks is the processing demand placed on the hippocampus during early stages of consolidation [54]. In line with this, results from the present study showed a substantial impairment in the group trained on the two spatial tasks and injected with muscimol into the ACC prior to the remote time point. These results support the hypothesis of a progressive strengthening of cortical connections as time progresses and an accelerated strengthening of cortical networks with increasing memory processing demands.

It should be emphasized that group WM/RAM:Remote:Muscimol showed no indication of an intact memory representation for the platform location. Multiple memory traces that are hippocampal-dependent, such as those arising from spatial tasks, may compete for hippocampal resources if they are encoded within a relatively short timeframe. In this situation, reliance upon cortical structures, such as the ACC, may more readily occur so that subsequent memory traces do not interfere with one another. Increased involvement of cortical regions may free hippocampal resources to process and encode a new memory trace. As many models suggest [4], [55], [56] a gradual shift in memory storage from the hippocampus to cortical regions is advantageous. Multiple memories relying heavily on hippocampal resources may accelerate the time course of this shift in an effort to free hippocampal resources. Future studies could manipulate the basic behavioural procedure outlined in this experiment to test the robustness of this hypothesis. For example, the order of training tasks could be reversed, or a RAM probe (instead of a WM probe) could be used. It would also be of interest to conduct the present experiment using a non-spatial task following WM training.

To accompany the behavioural findings, we examined c-Fos labeling patterns in the CA1 region of the hippocampus. Analyses revealed remotely tested groups had significantly higher numbers of c-Fos positive cells than recently tested groups. While these results are similar to other data [40], [54], [57], [58], they are not consistent with a gradual disengagement of the hippocampus over time (see [10], [39]). Rather, increased hippocampal activation at remote time points provides support for the multiple trace theory model, which suggests that spatial memories always require hippocampal function [59]–[62]. In this model, spatial memories can be represented in extra-hippocampal regions, but they lack detail and salience. As the WM task is a hippocampal-dependent task [63], [64], it comes as no surprise the hippocampus was activated during the probe test. From the current study, it is impossible to tell whether the increase in c-Fos positive cells resulted from increased activation of the hippocampus due to performance of the task (i.e., swimming and navigation), or, whether the increase resulted from a cognitive process (i.e., retrieving the stored memory).

Rudy and Wright-Hardesty [65] describe a theory that argues that the differential involvement of the prefrontal cortex in older memories reflects natural forgetting through decay or interference, resulting in older memories being weaker and more difficult to retrieve. In this model, weaker memories require additional activation from the prefrontal cortex for retrieval. Rudy and Wright-Hardesty also discuss a negative correlation between hippocampal and ACC activity, whereby high ACC activation occurs in response to inadequate activity or function of the hippocampus. The present study showed elevated hippocampal activity in the CA1 during the remote memory tests compared to retrieval of recent memories. In the alternative hypothesis [65], the remote spatial test would have resulted in increased cognitive processing due to natural decay or forgetting and thereby, required greater network activation. In the control case, increased CA1 hippocampal and ACC activity would be required during the remote time point to overcome the forgetting or decay that might occur. When the ACC was inactivated, the CA1 region would continue to be active but without concurrent activity in the ACC, the memory would not be retrieved properly.

A number of reports [40], [54], [58] used the water maze task as a behavioural measure and found increased activation of the hippocampus at remote compared to recent time points; consistent with the present results. Others used 1) a modified radial arm maze task that incorporated different start locations and retention testing in a single trial in an effort to incorporate aspects of a standard water maze procedure [57]; 2) contextual fear conditioning [39]; or 3) a five-arm maze [10]. Other studies have revealed similar levels of Fos expression following recent and remote probes on the water maze task [25]. Factors surrounding the task used such as complexity, cue saliency, and other environmental influences, as well as decay or forgetting, may result in a greater burden placed upon the hippocampus during remote memory retrieval. Remote hippocampal activation may result from a greater burden on hippocampal function resulting from a complex spatial environment and navigational demands on the WM task. The burden on hippocampal function may be less with more recent memories. Alternatively, increased hippocampal activation at remote time points might reflect relearning requirements following longer time intervals [57].

Interestingly, group WM/RAM:Remote:Muscimol (as well as WM Only:Remote:Muscimol and WM/RAM:Remote:Muscimol), similar to all other remotely probed groups, showed greater numbers of c-Fos positive cells than recently probed muscimol or saline groups, but showed impaired performance on the water maze probe test when the ACC was inactivated. As one interpretation of our findings (elevated c-Fos labeling in the CA1 in all remote groups, but no consistent behavioural evidence for an intact memory representation at the remote time point), we suggest that elevated activity in hippocampal networks is required for retrieving a remote spatial memory and that remote spatial memory is supported in networks in the ACC. Alternatively, it could be the case that the ACC is involved in remote memory retrieval from the hippocampus, and when offline, cannot perform this function. This interpretation would require an experimental design that allows for the testing of this hypothesis.

The findings presented here suggest continued involvement of the hippocampus in memory retrieval as well as involvement of the ACC. The pattern of behavioural findings suggests a progressive strengthening of cortical connections as time progresses and an accelerated involvement of the ACC in memory representation when hippocampal demand is taxed.

## Supporting Information

Data S1 **Raw data from the water maze probe test for all rats in each of the groups used in the present experiment.** To provide an accurate depiction of the search patterns exhibited by the different groups during the probe test, data were examined for the entire 60 second probe test and also parsed into two time bins: from 0–30 seconds and 31–60 seconds. Data in the first three columns show time spent in target quadrant from 0–30 secs (D), 31–60 secs (F) and 0–60 secs (H). Data in the next three columns show time spent in target area (counter; platform region) from 0–30 secs (K), 31–60 secs (M) and 0–60 secs (O). Data in the next three columns show target area crossings from 0–30 secs (R), 31–60 secs (T) and 0–60 secs (V).
(XLSX)
